# Various Wounds of Various Joints at Torbay Hospital, Torquay

**Published:** 1896-08-22

**Authors:** Arthur E. Watson

**Affiliations:** Resident Medical Officer, Torquay Hospital


### VARIOUS WOUNDS OF VARIOUS JOINTS AT TORBAY HOSPITAL, TORQUAY.

By Arthur E. Watson, M.R.C S., L.R.C.P., Resident Medical Officer, Torquay Hospital.

The wound of a joint, whether it be a large or small joint, is a frequent injury met with in hospital practice, and the success or failure of the treatment will in many cases depend on the care and attention of the surgeon, and in some cases much will depend on the nurse. The surgeon's object is, firstly, to promote healing of the wound by first intention; and, secondly, to obtain a moveable joint. We try to obtain the former by washing out the wounded joint with antiseptics, thoroughly cleaning the wound and using antiseptic dressing; the latter by employing passive movement and preventing the joint becoming fixed or ankylosed. I have purposely given examples of various kinds of wounds of joints—incised, lacerated, punctured wounds, and also of different joints affected. In case I. much of the credit was due to the nurses in their attention to detail and carrying out orders. In this case suppuration followed a wound of the knee-joint due to a second injury, and at one time it seemed almost hopeless to try to save the patient's leg; but constant fomentations of hot carbolic lotion, irrigating the joint with Lot. hydrag perchlor. several times a-day, keeping the limb at rest, and giving the patient a nourishing diet, happily resulted in a cure. Much of this treatment of necessity devolved on the nurses, and had it not been for their care and attention the patient would not now be able to earn his living and support a large family. We are now (owing to the antiseptic treatment of wounds) able to stitch up an ordinary incised wound of a joint from the first, but care should always be taken by the surgeon to syringe out the joint cavity thoroughly with an antiseptic, and in that manner get rid of all blood clot or foreign matter. In the days when antiseptics were not employed the wound of one of the larger joints was a most grave injury, suppuration often followed, and the unfortunate patient not unfrequently lost a limb or even his life. When the patient's health is unsatisfactory—in drunkards, patients suffering from Bright's disease, &c.—the results will not be so good, and the outlook is far graver. If the wounded joint is complicated by much laceration or bruising of soft parts, or if there be a fracture of one or more of the bones extending into the joint, amputation may be the only treatment. In the treatment of wounds of joints I would lay stress on the following: (1) The employment of strict antiseptic precautions; (2) keeping the joint at perfect rest; (3) attention to patient's general health; (4) passive movement at proper time; (5) not allowing the patient to use the limb too early. In the following cases no example of gunshot wound is given, this being fortunately rare in civil practice.

*Lacerated Wound of Knee Joint followed by a Second Injury — Suppuration — Recovery.*—A strong, well-developed man, thirty years of age, was admitted into the Torbay Hospital on May 19th, 1892. The patient, whilst working at a quarry, had been knocked over by a mass of rock, and had fallen some fifteen or twenty feet, his left knee striking against a sharp point of rock. On admission to hospital a jagged-looking wound one inch in length could be seen on the outer side of the left knee, and about two inches from the lower and outer margin of the patella. A copious sero-sanguineous discharge was oozing from the wound and could be pressed out from the under surface and side of the knee-joint. On introducing the finger into the wound it was found that the knee-joint had been opened. It was decided to enlarge the primary wound at once, and to make a counter-opening on the inner side of the joint. This was accordingly done, and a drainage-tube passed through, and the joint well irrigated with perchloride of mercury lotion (1 in 2,000). The limb was fixed on a back splint, and a dressing of blue wool, white wool, and carbolic gauze applied. The patient did well, and had only a very slight rise of temperature at night, the temperature falling to normal on the eighth day. There was only a slight serous discharge from the wounds for the first four days. The joint was irrigated twice daily with the mercurial lotion. One-half of the drainage-tube was removed on the eighth day, and the remainder five days later. The patient was discharged from the hospital on June 1st with the limb in plaster-of-paris, and was told to keep the foot off the ground and to walk with the aid of crutches. He came to the hospital several times, and although the left knee-joint was somewhat larger than the right, there was no increased heat or pain. On the morning of July 18th he was brought to the hospital complaining of great pain in the knee. He stated that the night before he tried to walk across his bedroom without crutches and had tripped and fallen heavily on the left leg. This caused intense pain, and he could not sleep. On examination the knee was found to be greatly swollen and tense; fluctuation could be obtained; it was extremely painful and tender. The skin round the joint was reddened, and the temperature was 102·2 deg. Fahr. He had had no rigors. He was readmitted to hospital, and on the afternoon of the same day a long exploring needle was thrust into the joint and a small quantity of thin pus withdrawn. The patient was then anæsthetised, and an incision made on each side of the joint, but more posteriorly than the first incisions. The joint was drained as before, and irrigated twice a day. Hot carbolic fomentations were applied every three hours, and he was given a sleeping draught at bedtime. The temperature continued raised for some weeks, being about 101 deg. in the evening, and falling to 99 deg., or normal, in the morning. The patient began to lose ground, and it was feared that amputation would be the only resource left. The discharge, however, lessened, and the temperature began to fall. On August 20th the following note was made: "The patient has much improved during the last fortnight. There is now a little serous discharge from the wounds. The knee, although still swollen, is not tender or painful to touch. His temperature has been normal since the 7th; he takes his food well and sleeps well." The patient continued to improve, and left the hospital on October 31st—fifteen weeks since admission. He has continued well ever since, and has for the last few months been following his usual work. The leg is straight; there is very little movement in the joint, but he can do a good day's work, and walk long distances without pain or fatigue.

*Incised Wound of Knee Joint.*—On May 7th a boy, aged 11, was admitted with an oblique wound 1½ ins. in length, extending downwards and inwards to the inner and upper border of the patella. The wound was a clean cut, and was caused by the patient falling on a piece of glass. On cleaning out blood clot, &c., the wound was found to extend into knee joint. After well irrigating the joint with Sol. hyd. perchloride, 1 in 4,000, and squeezing out all blood clots, the rent in the capsule was carefully stitched up with three fine silk sutures. Superficial and deep sutures were then passed through the skin and the surfaces of the wound brought into actual apposition. The wound healed by first intention, and patient was discharged cured on May 29th.

*Wound of Elbow Joint; Fracture of Olecranon Process.*—On April 25th a patient was admitted with a small wound, ½ in. in length, extending into the left elbow joint. There was also a fracture of the olecranon process. The joint was syringed out with Lot. hydrag. per., 1 in 4,000, and the arm put up on a straight splint, and the wound dressed antiseptically. The patient made an excellent recovery and has a very useful arm.

*Punctured Wound of Knee Joint with French Nail; the Nail Running Through the Patella.*—A healthy looking boy, aged 13, was admitted on May 18th, having fallen down and run a nail into the knee. On examination an ordinary French nail was found firmly fixed in the patella, and it was only by employing considerable force that it could be withdrawn. It was found that at least one inch of the nail had run into the patella, so it was surmised that the joint had been punctured. The wound was washed with antiseptic and dressed, and the joint kept at rest by fixing the leg on a back splint. The temperature rose next evening to 100·2, and the joint became swollen, hot, and tender. The skin wound suppurated slightly. Hot carbolic fomentations were applied every four hours, and three days after the temperature had fallen to normal, and the swelling, heat, and tenderness had markedly diminished. The boy is now quite recovered, although the joint is still kept at rest, and he has not left the hospital.

*Incised Wounds of Smaller Joints.*—Many cases of incised wounds of the smaller joints have been treated in the casualty out-patient department. In one case the metacarpo-phalangeal joints of the index and middle fingers, and also the first phalangeal joints of the ring and little fingers, were opened. The joints were thoroughly washed out with perchloride of mercury lotion, the wounds sutured, and hand placed on a splint and dressed antiseptically. Passive movement was commenced directly the wounds were healed, leaving the patient with useful and moveable fingers.

